# Comparison of Iran’s nursing education with developed and developing countries: a review on descriptive-comparative studies

**DOI:** 10.1186/s12912-022-00861-x

**Published:** 2022-05-06

**Authors:** Zahra Farsi, Morteza Nasiri, Seyedeh Azam Sajadi, Mohammad Khavasi

**Affiliations:** 1 Research Department and Community Health Department, Faculty of Nursing, Aja University of Medical Sciences, Tehran, Iran; 2 Student Research Committee, Shiraz University of Medical Sciences, Shiraz, Iran; 3 Operating Room Nursing Department, School of Nursing and Midwifery, Shiraz University of Medical Sciences, Shiraz, Iran; 4 Nursing Management Department, Faculty of Nursing, Aja University of Medical Sciences, Tehran, Iran; 5 Medical-Surgical Nursing Department, School of Nursing and Midwifery, Dezful University of Medical Sciences, Dezful, Iran

**Keywords:** Curriculum, Education, Iran, Nursing, Review

## Abstract

**Background:**

Iran’s nursing education has undergone significant modifications in the last decades, especially following the Islamic revolution and the Iran-Iraq war. This review outlined changing trends in Iran’s nursing education and evaluate its status compared to other developing and developed countries.

**Methods:**

Six international and two national electronic data sources were searched up to May 2021, using relevant keywords and terms. The studies were included if they addressed history, development, or evolutionary aspects of Iran’s nursing education or evaluated its status by comparing it with developing or developed countries, using Bereday's model. To obtain more relevant information, the organizational documents of the Iranian Ministry of Health and Medical Education and the Iranian Ministry of Culture and Higher Education were searched. Of 753 evidence found in the initial search, 73 were considered eligible for this review. A systematic and unbiased data synthesis was performed and a narrative and tabulated summary was presented.

**Results:**

The evolution in Iran’s nursing education has resulted in the establishment of Bachelor, Master, and Ph.D. programs. Iran’s nursing education system plays an important role in fulfilling the healthcare system’s mission, and it does not hold a dissatisfactory position in comparison with other developed and developing countries. However, this system is expected to be more versatile for the upcoming changes and advancements.

**Conclusion:**

Iran’s nursing education has a moderate rating despite recent changes. Hence, this system has to be modified in some aspects by adopting experiences of other countries, with an appropriate and successful education system, to prepare future highly competent nurses.

## Background

Nursing education, as a part of higher education system, plays an essential role in the training of nurses, who are known as the backbone of the primary healthcare system all over the world [1]. The nursing education is aimed to train qualified nurses with an appropriate level of knowledge, attitudes, and skills, who can promote the life quality of individuals, families, and community by accepting the roles and responsibilities in line with changing needs of society and developments of healthcare system [2, 3]. Following globalization and developing information and communication technology, nursing education has been developing in quality and quantity, especially in developing countries [4].

As with many other developing countries, nursing education in Iran was introduced by Western missionaries and is mostly influenced by education models in the United States and England [5]. However, in the last decades, especially following the Islamic revolution (1979) and the Iran-Iraq war (1980–88), nursing education in Iran has undergone significant changes and modifications to be more adaptable to the country’s context [6]. These changes were also made to address the growing complexity of the healthcare system, such as increase quality of care, promote nursing authority and competency, improve the social image of nursing profession, and evidence-based nursing care [7–9]. The modifications include but not limited to the establishment of nursing faculties, transferring of nursing education to the universities, gender equality in nursing education, and developing postgraduate programs [5, 10].

Despite the positive effects of educational changes on developing nursing profession in Iran, like many other developing countries, it has faced undeniable challenges in different aspects and levels. Some of these challenges include increasing rate of student admission to compensate for nursing shortage, inadequate faculty members and clinical instructors, inadequate facilities and clinical training settings, and the gap between knowledge and clinical practice [4, 11, 12].

In Iran, the most important challenge in nursing is the shortage of nurses [13]. The results of a study in 2015 in Iran indicate a shortage of 130,000 nurses and its forecast to 200,000 nurses in 2020 [14]. The number of nurses per hospital bed in different provinces of Iran is 0.6 (less than one nurse) to 1.5 people (more than one nurse). Also, the ratio of nurses per thousand population in Iran is 1.5 people. Iran ranks lower than Egypt, Turkey, Oman, UAE, Kuwait and Qatar [15]. The most important reasons for the challenge of shortage of nurses in Iran are poor social status, early retirement, immigration, willingness to abandon the current job, employment in other jobs, work-related injuries, low employment rates, housekeeping and increase of hospital beds [13]. One strategy considered in Iran to address the challenge of nursing shortage was to increase the number of nursing schools to increase nursing student enrollment. Every year, 11,000 to 15,000 people in Iran graduate from the field of nursing, and 50,000 nursing students are taught at three levels of bachelor, master and doctorate under the supervision of two thousand faculty members in Iranian universities [15]. Increasing the prevalence of chronic diseases is one consequence of life expectancy in Iran [16]. The High prevalence of chronic diseases and advancing age of the Iraniain population in recent years have been considered in the curricula of postgraduate programs and the field of geriatric nursing was launched in 2010 [17].

As a recent challenge, the Covid 19 pandemic has affected various aspects of Iranian life, including education. The results of a qualitative study in Iran showed that although the Covid 19 pandemic created many challenges in the nursing education process, these challenges were associated with opportunities such as emerging technology, innovative educational methods [18].

Considering this reason, and since high-quality education plays a pivotal role in training competent students that have a close relationship with quality of nursing care and patient’s safety, nursing education should move towards ongoing evaluation and promotion [19]. In the other word, it is necessary to modify the nursing education regularly and dynamically based on the ongoing economic, political, and social changes to progress the quality of nursing education and prepare nurses not only for the complexities of the modern health care system but also in meeting the health needs of the country [20].

Today, developing countries are trying to promote and reinforce their nursing education by adopting experiences of other countries, with appropriate and successful education systems [21]. Therefore, using the experiences of these countries in different education levels of nursing, by considering the cultural, political, economic, and social context of Iran, could help to resolve recent challenges in Iran’s nursing education [7]. Recent studies indicated a great interest in comparing the similarities and differences of nursing education in Iran and other countries at different levels to evaluate its status. However, to the best of our knowledge, these studies have not been synthesized to provide a comprehensive understanding of how to promote the nursing education of Iran. Hence, the review is to summarize the studies that evaluate the status of nursing education in Iran by comparing it with other countries, and then present strategies based on the synthesis of included studies. Also, to better describe the development and recent changes of nursing education in Iran, its related evolution was reviewed.

## Methods

A comprehensive computer-based search was conducted to find full-text and peer-reviewed studies published in English or Persian, up to May 2021. The search was performed on PubMed, Scopus, Web of Science Core Collection, ProQuest, Science Direct, Google Scholar, and also two Iranian databases, including Scientific Information Database (SID, http://www.sid.ir/) and MagIran (http://www.magiran.com) with no time restriction on the publication. A combination of the following keywords or terms was used: nursing, nurse, education, training, learning, teaching, instruction, coaching, curriculum, evolution, development, history, comparative analysis, Bereday's model, Iran, and Iranian. To find all qualified studies addressing the issue, a manual search was performed through the reference lists of the relevant bibliographies.

We included descriptive-comparative studies if it evaluated the nursing education system, programs, or curriculum in Iran, by comparing it with developing or developed countries using Bereday's model which composes four stages of description, interpretation, juxtaposition, and comparison [22]. We also included qualitative studies and reviews if they addressed history, development, or evolutionary aspects of Iran’s nursing education. But the evidence was excluded if it: 1) was conference proceeding, expert opinion or commentary, 2) only critiqued the Iranian nursing education without comparison with other countries, 3) used other models for comparison of the Iranian nursing education, 4) only reviewed the related challenges or issues in Iranian nursing education, and 5) had low rigor. The rigor of the evidence was evaluated according to methodological quality, authenticity, informational value, and representativeness on a two-point scale (high or low) by two independent investigators [23].

To better address the development process of Iran’s nursing education and understand the concern, organizational documents about Iran’s nursing education programs or curriculum were also included. These documents were obtained via electronic or manual search from the Supreme Council of Planning (SCP) affiliated with the Iranian Ministry of Culture and Higher Education or Supreme Council of Planning in Medical Science (SCPMS) affiliated with the Iranian Ministry of Health and Medical Education (MoHME).

**Fig. 1:**
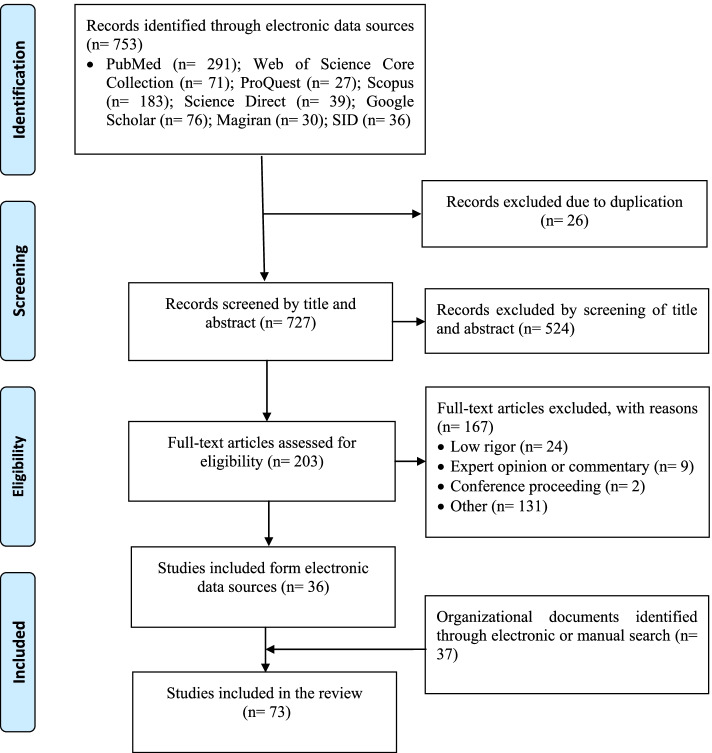
Flow diagram for identification of the evidence and selection process

The evidence was identified and selected by three investigators independently from November 11, 2019, to June 14, 2021. In the initial search, 753 studies were identified through the computer-based search. After removing 26 duplicates, 727 records were screened by titles and abstracts and 524 irrelevant records were excluded. Based on a full-text evaluation of the remaining 203 items, 167 records were excluded. We included 25 descriptive-comparative studies [17, 24–47], four qualitative studies [6, 48–50], and seven reviews [5, 7, 9, 10, 51–54] (Table 1). Also, we used 37 organizational documents [55–91]**(**Fig. 1**)**.

**Table 1:** Summary of the included descriptive-comparative studies on comparison of nursing education in Iran with developing or developed countries using the Beredy model

Program	**Authors, Publication year**	**Objective(s)**	**Main findings or conclusion**
Ph.D	[35]	Comparison of nursing Ph.D. curriculum in Iran and Toronto (University of Toronto)	The curriculum in two countries has focused on professional values, promoting community health, social justice, innovation, and student-centeredness; however, in Iran the focus has been on Islamic values and scientific development, and in Toronto emphasis has been placed on cultural values and the ability of international leadership. In the Iranian curriculum, unlike Toronto, most of the content of the courses is theoretical, and there is no clear relationship between the courses and the health needs of the community and students' needs and abilities. Contrary to the strict policy of publishing an article from a doctoral dissertation in Iran, this is not the case at the University of Toronto
	[34]	Comparison of nursing Ph.D. education program in Iran and Colombia School of Nursing	The curriculum in Iran has common points in course structure and characteristics with the curriculum in Colombia School of Nursing. Both programs are full-time and have a mission, perspective, and values base on strategic planning. Both curriculums have focused on researches and loser attention have paid to clinical education. In Colombia school of nursing, interview and research background are more important in the entrance exam
	[25]	Comparison of nursing Ph.D. education system and curriculum in Iran and Alberta School of Nursing	The program of both universities is based on the principles of strategic planning with mission, vision, and values. The curriculum in Iran coincides with the syllabus of the doctoral program of Alberta School of Nursing in some parts such as the profile and structure of the course. In Iran, student admission is subject to a master's degree but applications for admission to the University of Alberta are for individuals with a postgraduate degree in nursing and a degree in research statistics
	[43]	Comparison of nursing Ph.D. education system and curriculum in Iran and Toronto (University of Toronto)	Both programs are full-time, in-person, and student-centered. There are structural differences between the programs; but there are similarities during the educational period and the content of some courses such as research education, research proposals, and critique studies in Toronto, with the methodology of quantitative–qualitative research and critique of papers in Iran. Students' proposal writing course faces similar challenges. Toronto does not focus on the article extracted from the dissertation, but in Iran, it is considered necessary
	[38]	Comparison of nursing Ph.D. programs in Iran and Canada (universities of Alberta, McMaster, McGill, Ontario, and Victoria)	Both the countries have significant differences in fundamental elements of their curriculum, including mission, vision, aim, objectives, roles, and tasks of graduates. Also, academic units, final comprehensive exams, and research courses are different
	[30]	Comparison of nursing Ph.D. curriculum in Iran, Turkey, and Jordan (University of Jordan)	Regarding the characteristics and structure of the course among the three countries, there were many similarities. The only difference was that in Turkey, there were different branches in nursing and the possibility to enter the Ph.D. from the bachelor's degree. None of the countries uses advanced educational and telemedicine technologies
	[32]	Comparison of nursing Ph.D. education system and curriculum in Iran and John Hopkins School of Nursing	In John Hopkins Nursing School, educational plans are more various and students can choose the course units based on their need, thesis title, and supervisor's recommendation. Also, a significant relationship exists between the mission and goals of the curriculum in John Hopkins Nursing School. Some suggested that Iranian educational planners should be more careful in communicating the goals and structure of teaching in nursing Ph.D. education system
	[42]	Comparison of nursing Ph.D. curriculum in Iran and Widener University	The Widener University’s curriculum does not emphasize on writing papers as a course requirement; while it is obligatory in Iran to publish papers based on dissertations. In Iran’s nursing Ph.D. curriculum, the relationship between Islamic and professional values, and learners’ needs and capabilities are not clear. Also, the content of some courses is very abstract and emphasizes on theoretical issues. Courses are presented in both in-person and virtual classes at Widener University; however, they are presented only in in-person classes in Iran
	[36]	Comparison of the executive process of Iranian and British nursing doctoral dissertation	The Ph.D. dissertation in Iran mostly conduct in qualitative research and few researchers select mixed-method research; whereas in most British Universities, the dissertation is a quantitative research relevant to clinical settings. In both countries, it was mandatory to pass qualitative and quantitative research methods before obtaining a dissertation. Also, written and oral presentations of the project and its publication are similar
Master	[40]	Comparison of curriculum of masters of nursing management in Iranian universities and UC Davis University of California	Both programs showed similar approaches to theoretical and clinical teaching, except that peer learning, distance learning, and preceptorship were found at UC Davis University. The job duties mentioned for graduates in the US Davis Master's Degree Programs also indicate that the "Management- Leadership" tasks are similar in both programs, but given the educational content, the Iranian program was more practical and its main focus is management, leadership, and policymaking in clinical settings. However, at UC Davis University, nurses are prepared for leadership roles in the care system and faculty at colleges and specialist nurses in community
	[26]	Comparison of the education system and curriculum of masters of pediatric nursing in Iran and Toronto (University of Toronto)	The curriculum in both faculties is full-time, in-person, and emphasis on student-centered principal. At the nursing faculty of Toronto, more emphasis is placed on students’ creativity and students’ participation in education, and also evidence-based education and research are among the core elements of their curriculum. Many differences regard the structure of the course and admission conditions, but there are similarities in goals, roles, and abilities
	[46]	Comparison of education program of masters of emergency nursing in Iran and Australia (Flinders University)	Both countries have a mission, vision, and philosophy under the basic principles of strategic planning. The admission of the applicants in Australia is based on the individual's interest and attention to the individual's employment in the emergency department instead of exam which is important in Iran
	[28]	Comparison of education system of MSN in Iran and John Hopkins School of Nursing	The common aspects of the program in Iran and Johns Hopkins School of Nursing were attention to the needs of society, permanent changes, and diversity in nursing tendencies, choosing self-directed learning strategies, and pay attention to coverage and professional appearance. However, admission requirements, goals, philosophy, mission, vision, course content, the role of graduates, educational spaces, and administrative staff are different
	[47]	Comparison of curriculum of masters of neonatal nursing in Iran and Mcmaster University of Canada	The values and goals of both master programs are based on the beliefs and values of the community, and their mission is clear. The role of neonatal nurses in the Iranian program is reported as supportive, educational, research, and management; while the Canadian program does not mention the future roles of their neonatal nurses. Also, the McMaster University provides more comprehensive and more relevant admission requirements and postgraduate courses
	[29]	Comparison of MSN curriculum in Iran, Turkey (Florence Nightingale Faculty of Nursing), and Jordan (University of Jordan)	The programs in each country have special mission, goals, and job responsibilities. Admission requirements in Iran and Turkey include a BSN degree and admission to the entrance exam; an interview is also taking place in Turkey. However, in Jordan, volunteers must control the conditions, priorities, admission capacity, and plans at the faculty site and, if they are available, they can study at this course. In Turkey, it is also possible to pass this course as part-time and evening shifts
	[17]	Comparison of MSN curriculum in Iran and Japan (Chiba and Oita universities)	The educational system in Japan has different in some aspects including: admission interviews, English language requirements for admission, versatility and flexibility of the educational curriculum to facilitate student employment during education, integration of research-based units in the curriculum, and attention to community-based and home care nursing
	[44]	Comparison of curriculum of masters of geriatric nursing in Iran and Ireland (University of Dublin)	The main focus of program in Iran is on clinic, teaching, and research; and employment opportunities are too general. In addition, student admission is centralized and normative and tuition is free. In Ireland, job opportunities are more objective and proportionate to the objectives of the program; admission is done via a decentralized model and students should have selection criteria and tuition is not free
	[33]	Comparison of curriculum of masters of pediatric nursing in Iran and United States (John Hopkins School of Nursing)	Iran’s curriculum has distinctive philosophy, values, position, and career duties. The required qualification to enter the course is included having a BSN degree and passing the test. In the United States, interview and presenting the scores, curriculum vitae, and working licensure were essential. Pediatric nursing course is delivered during 4 semesters in Iran. In the curriculum of the United States, it is also possible to pass the course as part-time. The content of both curriculums is similar
Bachelor	[31]	Comparison of BSN curriculum among nursing schools of McMaster University of Canada, Hacettepe University of Turkey, and Tehran University of Iran	In the curriculum of Tehran and Hacettepe universities, no correlation was found between contents and educational goals, while a significant conformity was found between the theoretical and clinical goals of courses offered in the curriculum of McMaster University. The ability to transfer leadership, management, communication, critical thinking, and clinical decision-making skills were formally ignored in the curriculum of University of Tehran, while the mentors act just as a role model. There were programs and workshops for practicing critical thinking at Hecettepe University and the evidence-based nursing and research in nursing were stressed in educational goals; while the points were disregarded in the curriculum of the University of Tehran
	[24]	Comparison of BSN program in Iran and University of California, Los Angeles (UCLA)	The goals, missions, and structures of both programs are similar. Considering the characteristics and cultural diversity of patients, the patient-centered approach, caring for high quality and safe care are the goals of the UCLA Nursing School, which is less common in Iran. In UCLA Nursing School, clinical education is done in the simulator lab equipped with the most updated educational technology, but in Iran, it is conducted in a clinical setting, using modern educational approaches is less common in Iran. Graduates at UCLA may not work until getting a registered nurse license, but they work in Iran after graduation
	[27]	Comparison of BSN program in Iran and the International Islamic University of Malaysia	The length, number, and the majority of courses at the University of Iran and Malaysia are similar. However, there are differences between two universities in the goals, mission, and vision, admission system, and style of syllabus arrangement. In Iran, student admission focus on no interviews with governmental and non-governmental educational system; but in Malaysia, student enrollment is decentralized, with an interview and a non-governmental education system, accompanied by a social service project and a final project at the end of the course
	[39]	Comparison of systematic strategy in BSN of Iran and America (Johns Hopkins, Ohio, Seton Hall, Purdue, Chamberlain, Notre Dame universities), Canada (Western Ontario, Mount Royal universities), Australia (Queensland, Sydney universities)	A systematic strategy is more emphasized in American, Canadian, and Australian universities and using their experiences could help to promote the Iranian nursing education system in selecting students, effective teaching and learning process, and outcome
	[37]	Comparison BSN in Iran and George Washington University	Undergraduate nursing education in George Washington University has a two-year program, and student admission regulations are formulated independently by each. In Iran, undergraduate nursing education is a four-year program and has a semester system. The students are selected from among the candidates of experimental sciences through a nationwide university entrance examination. In all universities across Iran, one curriculum is presented, and there is limited flexibility in the program due to environmental conditions
	[45]	Comparison of BSN in Iran and Japan	Unlike Iran, the entrance to all levels in Japan is set by each university independently without passing the national test and admission criteria. Also, education programs in all levels and types and the number of units in Japan are selected by each university, whereas a similar program runs throughout Iran for all levels. Clinical training in Japan is presented by clinical educators, who are not affiliated with the School of Nursing, however, most nursing courses in Iran are taught by faculty of nursing
	[41]	Comparison of the curriculum of BSN in Iran and selected renowned universities in the world (Pennsylvania, McMaster, Florence, Edinburgh, Queensland, Rafik Hariri, Ras Al Khaimah, and Manipal)	Despite the many similarities in training and evaluation techniques of the examined nursing schools, many differences are observed depending on the level and purpose of the program in establishment and development of the contents and the method, extent, and type of addressing them. These differences create different content. However, the general objectives and educational contents in all curriculums are to some extent similar

*Abbreviations:BSN* Bachelor of Science in Nursing, *MSN* Master of Science in Nursing, *PhD* Doctor of Philosophy in Nursing

The data of all eligible evidence was summarized using a data extraction form. For all evidence, except organizational documents, details on the author(s) name, publication year, design and objective, evaluated program, and the main findings or conclusion were extracted. If it was necessary, contact was made with the author(s) of the selected evidence to elaborate on the required details. For organizational documents, characteristics of education program, publication year, and the main contents were extracted. With the data extraction, the data synthesis was performed. First, the included evidence was stratified by the evaluated program to organize data into a manageable framework. Second, in each stratify, relevant data of each source was extracted and compiled into a display matrix. Finally, the extracted data in each category were compared, and similar data were grouped and integrated into a comprehensive summation, and it was continually revised to be inclusive much [23]. For accuracy and confirmability of data extraction and synthesis, the process was independently performed by the three investigators, and discussions were held to reach an agreement.

## Results

### Characteristics of included evidence

Descriptive-comparative studies evaluated Bachelor of Science in Nursing (BSN) (*n* = 7), Master of Science in Nursing (MSN) (*n* = 9), and Doctor of Philosophy in Nursing (Ph.D.) (*n* = 9). These studies were published from 2014 to 2020 and most were in the Farsi, and only five were published in English [17, 31, 37, 38, 44] (Table 1). The remaining 11 review or qualitative studies addressed the history, development, or evolutionary aspects of Iran’s nursing education. These studies were published from 1968 to 2019 and all were in English, except one published in Farsi [5] (Table 2). Included governmental documents were published from 1982 to 2019.

**Table 2:** Summary of the included studies on the evolution of Iran’s nursing education

**Authors, Publication year**	**Objective(s)**	**Evidence type**	**Main findings or conclusion**
[5]	Overview of the evolutionary process of nursing in Iran	Literature review	Experimental nursing in Iran became a nursing academy with the effect education from the United States and England. With the onset of the Islamic revolution and war and increasing need, philosophy and nursing education models were changed. With the end of the war and the establishment of the Ministry of Health and Medical Education, the Nursing Organization and Nursing Board were founded in the areas for Iranian nursing education
[49]	Explaining the role of the Iran-Iraq war in the professionalism of nursing and medicine	Qualitative content analysis	“Training/Learning” was explored as a sub-theme of “promotion of personal and professional empowerment”. Most informants recognized the war as a constructive factor for nursing training and learning
[6]	Exploration of historical evolution of the Iranian nursing profession within a sociopolitical context	Historical study	Three major sociopolitical occurrences including the White Revolution, the Islamic Revolution, and the Iran-Iraq war had significant impacts on the Iranian nursing education
[7]	Overview on history and comparison of nursing education in Iran and China	Literature review	The major difference between two systems is validating the quality of nursing education graduates. In China, a national test is held to receive a certificate after graduation and before working as a nurse, but in Iran, there is no such program. The most common aspect between two systems is their educational paradigm concentrated on treatment of internal diseases and surgery with modeling the western biomedical model
[48]	Exploration of nursing developments in Iran during World Wars I & II	Historical study	The onset of academic nursing training in Iran was influenced by multiple factors such as war, famine, foreign forces, and the beginning of the modern age
[52]	Overview of history of nursing in the Islamic Republic of Iran	Literature review	Recent changes in Iranian nursing have resulted in university-based programs up to doctoral level
[92]	Overview of developing nursing education in Iran, within its economic and sociopolitical contexts	Literature review	Transformative changes in Iranian nursing education, specifically in higher education system, has resulted in nurses now undertaking study across all university-based programs up to doctoral level. Although these improvements in nursing education in Iran are to be applauded, much more attention is needed to ensure the competency of nurse practitioners in Iran
[9]	Overview of the professional nursing in Iran within its historical and sociocultural framework	Literature review	Nursing in Iran has progressed from the apprenticeship style of nurse training to the higher education sector, with the baccalaureate degree required for registered nurses. In Iran, as in other West and East Asian countries, the image of nurses has not changed despite advances in nursing practice, education, and research; necessitating professional socialization and policy changes
[50]	Exploration of gendered nursing education and practice among Iranian nursing students and faculties	Ethnography	Thematic analysis identified gender variations in care and compassion, spirituality, economic motives, and practice preference. Gendered nursing in Iran differentiates the nature and expression of caring between males and females and is embedded in the concepts of respect and modesty integrated into nursing teaching and practice. Iran’s support for male superiority over females is embedded in nursing education and practice
[54]	Overview on evolution of nursing education and profession in Iran within its religious, cultural, and political framework	Literature review	There are such basic issues as modes of education, cultural/religious states of consciousness, and the rights of women in Iran. These factors generally affect the development of the nursing profession and nursing education
[53]	Overview on nursing education in Iran	Literature review	Significant progress has been made 20 years in Iranian nursing education; however, there are problems to be solved

### Evolution of Iran’s nursing education

Iran's nursing education has undergone remarkable changes due to historical, religious, cultural, and economic factors from its beginning till now [9, 48, 54]. Significant evolution in Iran’s nursing education is summarized in Table 3. At first, women with no formal training as “Bimardar” cared for the patients in health centers (called “Darolshafa”) [7]. The American Presbyterian Missionary Society (APMS) began the training of a few nurses in a small missionary hospital in Urmia in 1915 [52]. One year later, the first nursing school was founded in Tabriz, where women with a high school diploma were enrolled in a three-year nursing education program [5]. In 1931, the Iranian Army Ground Force trained two types of nurses including Behyar (nurse assistant) and Komak-Behyar (associate nurse assistant). Behyar was recruited from the first-year high school students and the program consisted of a three-year curriculum. They can provide primary nursing care independently and more advanced care under supervision of a registered nurse (RN). The Komak-Behyar was trained during a hospital-based course conducted 2–6 months by RNs. In recent years, these nursing education programs are being eliminated from the nursing organizational structure [7].

**Table 3:** Overview of evolution in Iran’s nursing education

Topic	Year	City	Faculty/Institute
Start of basic nursing education	1881–1890	Tehran & Tabriz	
Establishment of the first nursing education by the American missionaries	1915	Uremia	Rezaeie (Uremia) Nursing School
Establishment of the first nursing school by religious group of Presbyterian (enrolment of women with a high school diploma in a three-year nursing program)	1916	Tabriz	Presbyterian, The Iranian State
Occupation of Tabriz by Ottoman forces and the transition of the nursing school to Hamadan	1918	Hamadan	
Formulation the Comprehensive Health Plan in which nursing education was considered	1921	Tehran	
Start of nursing education in Hamadan	1928	Hamadan	
Start of nursing education in Rasht	1931	Rasht	
Training of Behyar (nurse assistant)	1931	Tehran	Army Ground Force
Start of nursing education in Kermanshah	1932	Kermanshah	
Approval of the statute of nursing schools	1936	Tehran	The Iranian State
Recognition of nursing graduates and approval of Nurse Employment Law	1936	Tehran	The Ministry of Culture
Establishment of nursing schools in Tehran, Mashhad, Tabriz	1936	Tehran, Mashhad, Tabriz	The Iranian State
Establishment of nursing school in Shiraz	1937	Shiraz	The Iranian State
Approval of nursing school examination procedure	1938	Tehran	The Supreme Council of Education
Establishment of American Christian School of Nursing	1941	Mashhad	American Christian School of Nursing
Establishment of Oil Company Nursing School	1941	Abadan	Oil Company Nursing School
Approving amendment statute on nursing training	1943	Tehran	Population of Lion and Sun
Establishment of Reza Shah (Azadeh) Nursing School	1948	Tehran	Reza Shah (Azadeh) Nursing School
Establishment of Ashraf-e-Pahlavi (Tehran) Nursing School	1949	Tehran	Ashraf e Pahlavi (Tehran) Nursing School
Upgrade of nursing certificate to the nursing bachelor degree	1950	Tehran	
Establishment of Behyari School	1958	Tehran	Ministry of Health
Establishment of the first baccalaureate program	1965	Tehran	Firouzgar Higher Institute of Nursing
Training of behyar (nurse assistant)	1969	Tehran	Army Air Force
Establishment of the first military nursing school	1971	Tehran	NEZAJA Nursing School (Aja Nursing Faculty)
Establishment of the second military nursing school	1972	Tehran	Army Air Force
Approval of master’s program in two branches of “Nursing education” and “Nursing management”	1975	Tehran	National Nursing School
Establishment of master’s program in nursing education in five fields of “Medical-surgical nursing”, “Psychiatric nursing”, “Pediatric nursing”, “Community health nursing”, and “Nursing management”	1976	Tehran	National Nursing School
Establishment of master’s program in nursing education and management	1977	Tehran	Firouzgar Higher Institute of Nursing
Approval and establishment of two-year nursing program (Associate degree)	1982	Tehran	SCP, Imam Hussein University
Establishment of two-year discrete bachelor nursing program	1984	Tehran	National Nursing School
Revision of master’s program curriculum in nursing education	1986	Tehran	SCP
Revision of master’s program curriculum in nursing management	1986	Tehran	SCP
Reestablishment of bachelor program and revision of baccalaureate program curriculum	1987	Tehran	SCP
Revision of baccalaureate program curriculum	1992	Tehran	SCP
Establishment of Ph.D. program	1995	Tabriz	SCP
Revision of baccalaureate program curriculum	1995	Tehran	SCP
Revision of master’s program in nursing education	1995	Tehran	SCP
Revision of master’s program in nursing management	1995	Tehran	SCP
Revision of discrete bachelor program curriculum	1997	Tehran	SCP
Revision of Ph.D. program curriculum	2004	Tehran	SCPMS
Revision of baccalaureate program curriculum	2005	Tehran	SCPMS
Establishment of master’s program in critical care nursing	2008	Tehran	SCPMS
Establishment of master’s program in neonatal intensive care nursing	2009	Tehran	SCPMS
Establishment of master’s program in geriatric nursing	2010	Tehran	SCPMS
Establishment of master’s program in rehabilitation nursing	2010	Tehran	SCPMS
Establishment of master’s program in military nursing	2010	Tehran	SCPMS
Establishment of master’s program in emergency nursing	2013	Tehran	SCPMS
Establishment of master’s program in community health nursing	2013	Tehran	SCPMS
Establishment of master’s program in medical-surgical nursing	2013	Tehran	SCPMS
Establishment of master’s program in psychiatry nursing	2013	Tehran	SCPMS
Establishment of master’s program in pediatric nursing	2013	Tehran	SCPMS
Revision of baccalaureate program curriculum	2014	Tehran	SCPMS
Revision of Ph.D. program curriculum	2016	Tehran	SCPMS
Establishment of master’s program in nursing management	2017	Tehran	SCPMS
Establishment of master’s program in pediatric critical care nursing	2018	Tehran	SCPMS
Revision of master’s program curriculum in critical care nursing	2019	Tehran	SCPMS

*Abbreviations:SCP* Supreme Council of Planning affiliated with Iranian Ministry of Culture and Higher Education, *SCPMS* Supreme Council of Planning in Medical Science affiliated with Iranian Ministry of Health and Medical Education

The first four-year program as BSN was established in 1965 [5]. Then MSN was launched in 1976 with two main branches of “Nursing education” and “Nursing administration”. Gradually, the two branches were integrated as “Nursing” with different nursing fields [64, 66, 67, 71, 72, 76, 78–86, 89–91].

After the 1979 Islamic revolution in Iran, philosophy and nursing education models were changed [5]. The nursing education was moved from a hospital-based training model to an academic model and the gender-segregated health care system was established due to religious values [10, 52]. This system allowed male nurses to care for male patients and female nurses to attend to female patients, leading to male superiority over females in Iran’s nursing education [50, 54]. Likewise, with the onset of the Iran-Iraq war (1980–88), substantial changes were made in Iran’s nursing education [6, 48, 49]. The four-year nursing program changed to a two-year basic program as Associate's degree in nursing (ADN) in 1982 to accelerate care for soldiers and other victims in war zones and hospitals [63]. A two-year discrete bachelor program was established in 1984 for graduated nurses with ADN to continue their education [65, 74]. In 1987, the need for more nurses was reduced and the ADN program was canceled, and the four-year program was reestablished and its curriculum was reconstructed substantially [68]. The Iranian doctoral nursing program was introduced as Ph.D. in 1994 and the first program officially began in Tabriz University of Medical Sciences in 1995 [70].

During 1982–1997, the nursing programs were approved by the SCP. Since 2004, the nursing programs throughout Iran have been determined and supervised by the SCPMS. Iran’s nursing education includes three programs as BSN, MSN, and Ph.D. in both public and private universities, all supervised by MoHME. The public university system is free and provides equal opportunity for students, regardless of his/her income level. But in the private university system, called the Islamic Azad University, paying tuition is a must [7].

### Evaluation of Iran’s nursing education in different levels

#### Bachelor of Science in Nursing (BSN)

Iran’s BSN program was established in 1965 and it was reestablished and the curriculum was revised step by step in 1987, 1992, 1995, 2005, and 2014 [68, 69, 73, 75, 87]. The BSN program is the first level of academic nursing education in Iran, and passing this course is mandatory to reach RN [87].

The included studies compared the BSN program in Iran and some developed and developing countries, including United states (Johns Hopkins, Ohio, Seton Hall, Purdue, Chamberlain, Notre Dame, George Washington, Pennsylvania, and UCLA Universities) [24, 37, 39, 41], Canada (Western Ontario, Mount Royal, and McMaster Universities) [31, 39, 41], England (Florence University) [41], Scotland (Edinburgh University) [41], Turkey (Hacettepe University) [31], Australia (Queensland and Sydney Universities) [39, 41], Lebanon (Rafik Hariri University) [41], United Arab Emirates (Ras Al Khaimah University) [41], Malaysia (International Islamic University) [27], Japan [45], and India (Manipal University) [41]. The studies addressed differences in some components including philosophy, vision, mission, objectives, admission requirements and graduation, and curriculum (Table 1).

##### Philosophy, vision, mission, objectives

The philosophy of BSN in Iran is more on Islamic culture and spirituality and ethical considerations [87], while in most evaluated countries the focus is on training nurses who act according to theory and research, by considering the supreme values, society, social justice, honesty, and creativity. Likewise, vision and mission are wider in other countries with emphasis to address the local, national, and international needs. A combination of altruism, independence, integrity, social justice, and respect to various cultures and human dignity is addressed in other countries, considering critical thinking, clinical decision-making, and the skills required for comprehensive, decent, and evidence-based care for the patients [24, 27, 31, 37, 41, 45]. In Iran, belief and commitment, compassion, kindness, professional ethics, and effective professional communications are more addressed [87].

The objective of BSN in Iran is the training of nurses specializing in theoretical knowledge and practical skills to provide different services (healthcare, educational, research, counseling, managerial, support, and rehabilitation) to supply, maintain, and promote individuals, families, and community health, based on safety and quality [87]. These issues are described more completely in other reviewed countries. Likewise, other countries focus on critical thinking, analysis, communications, and cultural variation and aimed to enable students to take responsibility for leadership positions and evidence-based action [24, 27, 31, 37, 41, 45].

##### Admission requirements and graduation

Although applicants’ enrollment in most countries is decentralized by considering the prerequisite courses of BSN, the admission system in Iran has no focus on interview in both public and private systems and is based on high school graduates ranking in the Iran's national university entrance examination (Konkoor) and the nationwide regulations [24, 27, 31, 37, 39, 41, 45]. However, some Iranian universities have compulsory interviews focusing on applicants’ physical and mental health [87].

Upon successful completion of BSN program in Iran, graduates are awarded the degree and granted RN status and they are eligible to work in all wards of a hospital [87]. On the contrary to other countries that hold a national test for graduates to receive a certificate after graduation and before initiating the work as a nurse, validating the quality of nursing education amongst Iranian graduates is merely receiving their degree [24, 27, 31, 37, 39, 41, 45]. However, due to mandatory regulation by MoHEM, graduates must work in public hospitals for at least 24 months, and then they may apply for employment elsewhere [87]. In addition, once formally employed in any governmental hospital in Iran, nurses are guaranteed a job for 30 years [9]. However continuing education programs for nurses are provided and their competencies are evaluated annually by their managers [45]. Currently, about 200,000 nurses at various levels of nursing in Iran are providing services in hospitals and medical centers under the auspices of the Ministry of Health, the Social Security Organization, hospitals affiliated to the Armed Forces and the private sector, of which about 140,000 in the Ministry of Health Provide service [15].

##### ***Curriculum***

Even though nursing curriculum flexibility, style of syllabus arrangement, and numbers of units are different in Iran in comparison with other reviewed countries, to some extent there is a similarity in the length of the program, titles of units, educational spaces and areas, educational methods and techniques, and assessment methods [24, 27, 31, 37, 39, 41, 45].

Similar to most countries, the Iranian BSN is a four-year program with 8 semesters. Each academic year in Iran consists of two semesters: the first semester begins in the late September and ends in late January, and the second semester begins in February and ends in June. On the contrary to other countries, all Iranian universities must follow a basic curriculum established by the MoHEM, although some minor flexibility is allowed within the predetermined curriculum [87].

The Iranian BSN program consists of 130 units, which includes 22 general, 15 basic sciences, 54 core, 18 clinical training, and 21 field apprenticeship. Besides these mandatory units, students should participate in some workshops. Core units in Iran include “Nursing care for adults and the elderly”, “Maternal and newborn health”, “Pediatric nursing”, “Psychiatric nursing”, and “Community health nursing” [87]. The number of units in the curriculum of other courtesies is much more than that of Iran, while the number of clinical training and field apprenticeship units in Iran is much more than that of other courtiers. In other countries, students may also choose more than units, including research on a specific nursing problem. Depside other countries, no clear conformity was found between educational units and philosophy, vision, mission, and educational objectives in Iran because most are drawn from the treatment-centered view [24, 27, 31, 37, 39, 41, 45].

Similar to most countries, the Iranian students learn the theoretical principles of basic nursing skills in the theoretical classes and practical skills in the clinical skill centers and laboratories (biochemistry, physiology, and microbiology). The clinical training process in Iran is arranged from simple to difficult and takes place during patients’ care based on the nursing process. Students begin their clinical training from the second semester and this is run concurrently with theoretical courses until the end of the third year. In the fourth year, students participate in full-time hospital-based education apprenticeship. During clinical education, students can work with patients in various departments of general hospitals (internal-surgical, pediatric, obstetrics and gynecology, psychiatry, emergency, and critical care) [87].

Depside other countries that special attention is paid to cooperation and health of clients, family, and society and considering urban and rural healthcare centers and the community as educational spaces and areas, prevention type I and III have been little considered in Iran’s nursing due to the dominance of treatment-centered view in Iranian nursing. Clinical training in other countries usually presents by a Doctor of Nursing Practice (DNP), while an informal “preceptorship educational model” is dominant in most of the Iranian faculties due to inadequate faculty members, in which students will be trained under the supervision of expert nursing staff or master level students, who may not have sufficient teaching skills. However, similar to other countries, the core units in Iran are usually taught by faculty members, who generally have an MSN or Ph.D. degree [24, 27, 31, 37, 39, 41, 45].

Nursing education in the BSN of Iran is a mutual process and is based on the interaction of the trainer-trainee to achieve educational objectives. A wide range of methods and techniques is listed in the Iranian curriculum far from the actual methods. These methods include conferences, seminars, small group discussions, educational workshops, case presentation, journal club, healthcare and morning report, working and educational rounds, use of e-learning methods, cooperation in training of the lower ranks, and self-education and self-study. Of these, case presentation is the main teaching strategy in clinical settings, especially in intensive care units [87]. The educational methods and techniques applied in other countries including but not limited to learner feedback, class observation, computer classes, use of educational technologies (i.e., PowerPoint), group projects, student self-learning, conferences, e-learning, and professional workbooks [24, 27, 31, 37, 39, 41, 45].

Similar to other countries, formative and summative assessment is suggested in Iran through written and oral examinations, interactive computer examination, objective structured clinical examination, objective structured field examination, direct observation of procedural skills, 360-degree appraisal test, portfolio assessment, logbook completion, clinical assignment assessment, and use of a checklist for performance assessment [87]. However, there is no definite trend for assessment of clinical courses and field apprenticeships in reality, and assessment is mostly done personally. In most reviewed countries, self-assessment is emphasized and there is a coordination committee to supervise students’ achievement, and it is possible for weak students to get private counseling and teaching [24, 27, 31, 37, 39, 41, 45].

#### Master of Science in Nursing (MSN)

From the three nursing programs in Iran, the modifications in the MSN program have been more noticeable. This program was launched with two main branches of “Nursing education” and “Nursing management” 1975–77. Gradually, the two branches were integrated as “Nursing” with different nursing fields to make nursing more specialized and increase the quality of nursing services. Currently, there are different nursing Master's fields in Iran including: “Medical-surgical nursing”, “Psychiatric nursing”, “Pediatric nursing”, “Community health nursing”, “Critical care nursing”, “Neonatal intensive care nursing”, “Military nursing”, “Rehabilitation nursing”, “Emergency nursing”, “Geriatric nursing”, and “Nursing management”. Also, recently another field was approved as “Pediatric critical care nursing [64, 66, 67, 71, 72, 76, 78–86, 89–91].

The included studies compared the different fields of MSN in Iranian universities and UC Davis University of California [40], University of Toronto [26], Flinders University [46], John Hopkins School of Nursing [28, 33], Mcmaster University of Canada [47], Florence Nightingale Faculty of Nursing [29], University of Jordan [29], Chiba and Oita universities [17], and University of Dublin [44]. The Iranian MSN curriculum in most felids was modified in recent years to address the ongoing changes [64, 66, 67, 71, 72, 76, 78–86, 89–91]. Based on the included studies, noticeable similarities are addressed in some components of MSN in Iran and other universities; however, the admission requirements and roles of graduates are still different (Table 1).

##### Philosophy, vision, mission, objectives

The same as other universities, the MSN in Iran has a philosophy, mission, and vision under the basic principles of strategic planning and based on the beliefs and values of the community. However, these components are much clear in other universities. To some extent to other universities, the objective of MSN in Iran is to improve the level of knowledge, skills, and competency of graduates at a higher level than the BSN in different nursing domains. The main focus of the program in Iran is on clinic, teaching, and research; while in other universities, more attention is paid to the needs of society, permanent changes, and diversity in nursing tendencies [17, 26, 28, 29, 33, 40, 44, 46, 47].

##### Admission requirements and graduation

The MSN program is held in most public universities and also in some private universities in Iran, with identical criteria and admission exams. Although admission in most universities is done via a decentralized model by considering interview, curriculum vitae, and working licensure, the admission system in Iranian public and private systems is centralized with no focus on the interview. Also, tuition is free in most Iranian public universities, while tuition is not free in other countries [17, 26, 28, 29, 33, 40, 44, 46, 47].

In Iran, applicants can enroll in an MSN program in their favorite fields, if they can meet these criteria: 1) having a BSN degree from international or national universities approved by the MoHME with a grade point average ≥ 14 out of 20; 2) having at least two years’ work experience at clinical centers for enrollment in master's programs of “Critical care nursing”, “Neonatal intensive care nursing”, “Emergency nursing”, and “Nursing management”; and 3) obtaining at least 50% of the total average score on the annual national entrance exam (also obtaining at least 30% of the total score of the annual national entrance exam). The exam items are similar in all nursing fields and the only difference is in their coefficients. These items include “Medical-surgical nursing”, “Maternal and newborn health”, “Pediatric nursing”, “Psychiatric nursing”, “Community health nursing”, and “English language” [62].

Graduates of the MSN in Iran mainly become in-charge of medical sections; however, they have opportunities in the universities to act as a researcher or instructor. The job duties mentioned for graduates in Iran are too general, while they are more objective and proportionate to the objectives of the program in other countries [17, 26, 28, 29, 33, 40, 44, 46, 47].

##### Curriculum

The Iranian MSN program is 4–6 semesters in all fields. The total is 32, including 28 basic or/and core units and 4 thesis units. Also, applicants must participate in workshops based on their selected fields. The courses are different based on specific fields; however, most courses focus on theoretical knowledge and clinical skills in nursing care. In all fields, applicants learn quantitative research methodology and statistical analyzes. Seminar and thesis courses are usually presented in the second and third semesters, respectively. The seminar course focuses on the issues and challenges of nursing [78–86, 89, 91].

Similar to most countries, the Iranian program is full-time and emphasizes student-centered principles. The course content in Iran is more practical and its main focus is management, leadership, and policymaking in clinical settings, while at other universities content is considered to prepare nurses for faculty at colleges and specialist nurses in community. Also, in other countries, versatility and flexibility of the curriculum are more notable and an emphasis is on students’ creativity, students’ participation in education, evidence-based education, and research. The MSN in Iran and most of reviewed universities has similar approaches to theoretical and clinical teaching; however, peer learning, distance learning, and preceptorship are more highlight in other universities [17, 26, 28, 29, 33, 40, 44, 46, 47].

#### Doctor of Philosophy in Nursing (Ph.D.)

The doctoral nursing program in Iran was introduced as Ph.D. in 1994, and the curriculum underwent changes in 2008 and 2016 [70, 77, 88]. The number of faculties that present this program has increased significantly in recent years and the program is currently held in most high-rank public universities [60].

The included studies compared the Ph.D. program in Iran and Canada (Toronto, Alberta, McMaster, McGill, Ontario, and Victoria universities) [25, 35, 38, 43], the United States (John Hopkins School of Nursing, Colombia School of Nursing, and Widener University) [32, 34, 42], England [36], Turkey [30], and Jordan (University of Jordan) [30]. The included studies mentioned differences in the admission requirements and curriculum (Table 1).

##### Philosophy, vision, mission, objectives

The program in Iran is based on the principles of strategic planning with philosophy, vision, and mission. The Ph.D. program in Iran was modified in 2016 based on Iranian culture and beliefs with more focus on clinical education and setting [88]. One strength of this modification was reported to be the alignment of the values and beliefs of the program based on the Islamic values system and gaining competencies such as critical thinking, clinical argumentation, problem-solving, evidence-based decision making, and recording an analytical report [25]. The program objective in Iran is to prepare graduates as teachers with educational and research competencies, and improve their intellectual and creative abilities to develop nursing knowledge. The Ph.D. graduates, as knowledgeable professionals, are expected to provide valuable insights into nursing issues and promote the quality of nursing care [88]. The philosophy, vision, mission, and objectives in other countries seem similar to Iran [25, 30, 32, 34, 35, 38, 42, 43].

##### Admission requirements

In Iran, graduates of MSN who are admitted at both the annual national entrance exam and scientific interview can enroll in the Ph.D. program [61]. Although the interview is a common criterion in Iran and other countries, the interview method is to some extent different in Iran. Also, there is a possibility in some countries to enter the Ph.D. from the BSN degree, while in Iran only graduates of MSN could be admitted [25, 30, 32, 34, 35, 38, 42, 43].

Depside in other countries that interview and research background are more important in the entrance exam, the focus in Iran is on both exam and interview. The proportion of the exam and interview was 70% and 30% until 2016 [56]. However, the exam and interview each account for 50% of the total score since 2017. The exam is conducted by the MoHME and its items until 2017 were “Nursing theories and their application in nursing”, “Management theories and their application in nursing”, “Educational theories and their application in nursing”, and “Statistics and research methodology in nursing” [57]. However, the content of the exam was revised in 2018 and currently includes “Statistics and research methodology”, “Fundamentals of nursing care”, and “Educational talent” [59]. The interview until 2016 was performed in one station by the selected faculty members of universities that admit Ph.D. students [55]. However, since 2017 interviews are conducted by the same experts during an objective structured clinical examination with six stations including: “Presentation of master's thesis and its related article”, “Presentation of scientific contents”, “Proficiency in English language”, “Search in scientific databases”, “Proficiency in clinical nursing”, and “Proficiency in nursing challenges” [58]. Also, applicants are assessed during the interview for their educational, research, and technology backgrounds, which account for 20% of the total interview score [60]. Applicants must have an English certificate with the appropriate score, approved by the Iranian SCPMS [61].

##### Curriculum

Duration of the Ph.D. program in Iran is 4–5 years, consisting of 45 units including 25 units for the theoretical courses (19 core and 6 non-core units) and 20 units for the dissertation. Since 2017, students must pass 6 non-core units under their dissertation or field of master’s degree with approving their supervisor and Graduate Council. The main theoretical courses of this program include “Philosophy of science and nursing”, “Theorizing in nursing”, “Management and leadership in educational nursing”, “Quantitative and qualitative researches methodology”, “Advance inferential statistics”, “Mixed-methods studies methodology and instrument development”, “Management, leadership, and policy-making in nursing”, “Educational systems in nursing”, and “Challenges and issues in nursing”, which all present with a critical approach [88].

After passing all theoretical courses, students must pass a comprehensive exam to start their dissertation in a research course. The dissertation must be conducted in the nursing discipline after approval of the supervisor and the Educational Council or the Graduate Council [88]. Students may select instrument development or one of the qualitative research designs (grounded theory, phenomenology, action research, historical research, and ethnography) for their dissertation [36]. However, there is a great interest in mixed-methods studies in recent years (Rafati et al., 2015).

The nursing Ph.D. course in Iran is similar to that of other countries in some aspects. The same as most countries, the program in Iran is full-time and student-centered; however, working hours are less in Iran. Also, there are similarities in the educational period and the content of some courses such as research education and critique of papers. In most countries, courses are presented in both in-person and virtual classes; however, they are presented only in in-person classes in most Iranian universities. Despite other countries that the Ph.D. curriculum is more various, in Iran students have not the ability to choose the course units based on their need, dissertation, and supervisor's recommendation. In the Iranian curriculum, unlike other countries, the content of most courses is very abstract or emphasizes theoretical issues, and there is no clear relationship between the courses and the health needs of the community and students' needs and abilities. Likewise, the content of some courses in the Iranian program has overlap with some courses in the MSN program. The Ph.D. dissertation in Iran mostly conduct in qualitative research and few students select mixed-method research; whereas, in most other universities, the dissertation is a quantitative research relevant to clinical settings. In Iran, the students must publish at least an article from their dissertation in high-quality journals indexed in Scopus besides Pubmed or ISI web of Sciences; whereas in other countries, there are less focus on this issue [25, 30, 32, 34–36, 38, 42, 43].

The Ph.D. program in Iran was modified to be more focused on clinical education and setting, but there is no clear relationship between the mission and goals of the curriculum [88]. The current program is so different from the doctor of nursing practice (DNP) program because no determined positions are provided in Iranian clinical settings for graduates and they will likely work the same as previous graduates as faculty members at universities and educational or/and research centers. Hence, the last modified program seems to need revision and reconsidering based on the experiences of the advanced countries and the needs of the Iranian community with a multi and interdisciplinary partnership and cooperation model, specifically in the content of courses and the resources [25, 30, 32, 34–36, 38, 42, 43].

## Discussion

Nurses are the largest group of healthcare providers who play an important role in fulfilling health system mission. Studies have emphasized on the key role of nurses in patients’ lifestyle improvement, diseases prevention, health promotion, and patients’ coping with chronic diseases [93]. In Iran, patients’ level of awareness and expectations about nursing quality care has increased in recent years, which highlight the importance of nurses with more desirable professional competencies [9, 94].

The role of healthcare professionals, including nurses, is expected to change soon. Conventional models of healthcare that mainly focus on providing emergency, acute, and elective services are imperative, but it is not enough, since it cannot provide integrative and collaborative care. Also, sharing information is required for future’s patients, who will become elderly, multiple morbidities, dependent, and often confused [95].

Considering the recent changes and challenges of the healthcare system and educational system, nursing education in Iran requires more attention. Based on the included studies, the Iran’s nursing education does not hold a dissatisfactory position in comparison with other countries. However, Iranian nursing educational system and programs has to be reviewed and revised in selecting its goals and graduate needs, especially in postgraduate levels. We suggest that the concept of “hospitals without walls” to be given more attention in the training of nursing students to provide care at a place and time that will make the patient or client feel more comfortable with emphasis on holistic, integrative, and collaborative care, using a multidisciplinary team approach [95]. Providing care in the community and for elderly and patients involved in multiple chronic diseases should be prioritized, which needs more attention of the Iranian nursing educational system. Even though MSN program in “Geriatric nursing” and also “community health nursing” was developed, there is no certain work position for graduates, and most work in other fields of nursing [44, 81, 84]. In addition, “Telenursing” education should be embedded in the Iranian nursing curriculum because the concept of “Telenursing” will be more emphasis in the upcoming years in Iran and most services are expected to be provided in this way. Nursing students must be trained in a way to provide care in high-tech work environments by providing the required infrastructure and equipment [96]. Generally, the goals of Iran’s nursing education should addressed: 1) advancement in technology; 2) globalization; 3) the era of the educated consumer, alternative therapies, genomics, and palliative care; 4) a shift to population-based care and the increasing complexity of patient care; 5) healthcare costs and challenges posed by managed care; 6) a growing need for interdisciplinary education and collaborative practice; 7) the impact of health policy and regulation; 8) the growing nursing shortage along with the opportunities for lifelong learning and workforce development; and 10) significant advances in nursing science and research [97].

Most Iranian universities or educational nursing programs do not have a clear philosophy, mission, and goals to develop their framework. In addition, Iranian nursing educational programs, especially in graduate level, consist mainly theoretical lessons, which are usually not relate to each other and does not consider the professional interests of students and the community’s needs and demands. Iranian postgraduates’ programs often result in some impracticable thesis/dissertation, while the students do not acquire enough skills and expertise in a specialized nursing field. Hence, Iranian nursing educational system and programs has to be reviewed and revised in different dimensions. In this regards, most included studies have suggested a decentralized method for admitting applicants and a need for more flexible curriculum based on the needs of the community and common diseases while considering cultural, social, historical, and economical background [17, 24–47]. In addition, these suggestions should be prioritized:

- Training nursing students in “Community health nursing” and “Geriatric nursing”
- Preparing nursing students to work in high-tech work environments
- Promoting nursing students' communication skills to provide multidisciplinary care for patients and their families
- Motivating nursing students for self-preparation to provide care for patients in ever-changing healthcare systems

## Conclusion

The Iranian nursing education system does not hold a dissatisfactory position. However, to prepare future highly competent nurses to be more versatile for the upcoming changes and advancement, it is expected to Iranian nursing education be reviewed and revised in selecting its goals; admission methods; graduate needs; and research, teaching, and evaluation methodologies, by adopting experiences of other countries, with an appropriate and successful education system.

## Data Availability

The datasets used and analyzed during the present study are available from the corresponding author on reasonable request.
